# A Shortest Distance Priority UAV Path Planning Algorithm for Precision Agriculture

**DOI:** 10.3390/s24237514

**Published:** 2024-11-25

**Authors:** Guoqing Zhang, Jiandong Liu, Wei Luo, Yongxiang Zhao, Ruiyin Tang, Keyu Mei, Penggang Wang

**Affiliations:** 1 North China Institute of Aerospace Engineering, School of Remote Sensing and Information Engineering, Langfang 065000, China; zhanggq@stumail.nciae.edu.cn (G.Z.); liujd@stumail.nciae.edu.cn (J.L.); zhaoyx@stumail.nciae.edu.cn (Y.Z.); trytry@nciae.edu.cn (R.T.); 18130679197@163.com (K.M.); wangpg@stumail.nciae.edu.cn (P.W.); 2 Aerospace Remote Sensing Information Processing and Application Collaborative Innovation Center of Hebei Province, Langfang 065000, China; 3 National Joint Engineering Research Center of Space Remote Sensing Information Application Technology, Langfang 065000, China; 4 North China Institute of Aerospace Engineering, School of Aeronautics and Astronautics, Langfang 065000, China

**Keywords:** precision agriculture, UAV, Q-learning, deep neural network, shortest distance prioritization, root mean square propagation

## Abstract

Unmanned aerial vehicles (UAVs) have made significant advances in autonomous sensing, particularly in the field of precision agriculture. Effective path planning is critical for autonomous navigation in large orchards to ensure that UAVs are able to recognize the optimal route between the start and end points. When UAVs perform tasks such as crop protection, monitoring, and data collection in orchard environments, they must be able to adapt to dynamic conditions. To address these challenges, this study proposes an enhanced Q-learning algorithm designed to optimize UAV path planning by combining static and dynamic obstacle avoidance features. A shortest distance priority (SDP) strategy is integrated into the learning process to minimize the distance the UAV must travel to reach the target. In addition, the root mean square propagation (RMSP) method is used to dynamically adjust the learning rate according to gradient changes, which accelerates the learning process and improves path planning efficiency. In this study, firstly, the proposed method was compared with state-of-the-art path planning techniques (including A-star, Dijkstra, and traditional Q-learning) in terms of learning time and path length through a grid-based 2D simulation environment. The results showed that the proposed method significantly improved performance compared to existing methods. In addition, 3D simulation experiments were conducted in the AirSim virtual environment. Due to the complexity of the 3D state, a deep neural network was used to calculate the Q-value based on the proposed algorithm. The results indicate that the proposed method can achieve the shortest path planning and obstacle avoidance operations in an orchard 3D simulation environment. Therefore, drones equipped with this algorithm are expected to make outstanding contributions to the development of precision agriculture through intelligent navigation and obstacle avoidance.

## 1. Introduction

Agriculture has been a pivotal industry throughout human history [1]. Precision agriculture represents a significant evolution from traditional farming systems, leveraging modern technologies to enhance crop yields while reducing resource use [2]. In recent years, UAVs have gained widespread adoption in agriculture due to their high flexibility, mobility, low risk, and cost-effectiveness. For instance, UAVs enable precise spraying through route planning, ensuring the uniform application of agrochemicals to crops [3]; collect crop growth data to support decision-making and improve management strategies [4]; and monitor soil moisture and crop conditions to guide precision irrigation [5]. However, UAVs encounter challenges in agricultural applications due to the large-scale and complex environments, with the presence of hazardous areas and obstacles elevating mission costs and risks. By employing effective route planning, obstacle avoidance capabilities can be enhanced, flight costs can be reduced, and agricultural tasks can be completed efficiently.

**Figure 1:**
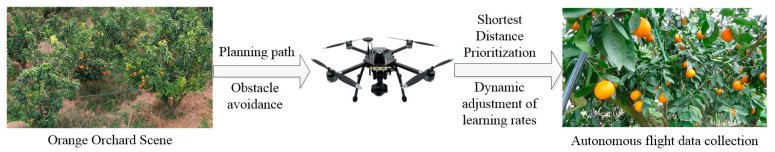
Orangery UAV collecting data.

With the integration of artificial intelligence (AI) [6], the Internet of Things (IoT) [7], and computer vision technologies [8], UAVs have gained access to new application layers that enable the automation of inspection processes and the generation of data to support decision-making, such as for selective pesticide application and reduction. In autonomous inspection and monitoring tasks, UAVs must determine optimal, or near-optimal, collision-free paths and target locations, requiring constant monitoring of the vehicle during operation. In dynamic environments, when sensors detect unforeseen obstacles, the path planning system must quickly respond to recalculate the assigned route, ensuring obstacle avoidance until the UAV reaches its final destination [9]. This study aims to propose an optimal path planning algorithm that enables UAVs to autonomously perform data collection tasks in orange groves, thereby reducing labor costs and utilizing the collected data for subsequent yield estimation, as illustrated in Figure 1.

Agricultural UAV trajectory planning focuses on designing optimal, safe, and accurate flight routes for UAVs to operate effectively in farmland environments, guiding them from the starting point to the completion of their tasks. The A-star algorithm [10], an advanced adaptation of Dijkstra’s algorithm [11], incorporates a heuristic function [12] to improve computational speed. Despite these enhancements, it tends to produce routes with numerous turns, reduced smoothness, and limited safety margins [13]. The Rapid Exploration Random Tree (RRT) algorithm [14], which bases its path planning on random sampling, is distinguished by its robust search capacity and rapid execution. Yet, this randomness sometimes leads to routes that are excessively long and lack smoothness due to the frequent inclusions of many redundant turns. The Artificial Potential Field (APF) algorithm [15] is one of the most popular algorithms among the real-time path planning algorithms because of its efficiency and is frequently used together with other planning algorithms. However, it sometimes falls prey to a local optima issue, which negatively affects its performance. In contrast, increasingly complex optimization approaches like the Ant Colony Algorithm (ACA) [16], Genetic Algorithm (GA) [17], and Particle Swarm Optimization (PSO) denote a transition from localized intelligence to the population model. These methods are really good at solving tough navigation problems, but they could be slowed down by being computationally expensive, convergence being gradual, and the possibility of getting stuck in a local minimum.

Reinforcement learning (RL) is a significant branch of machine learning, spanning across various disciplines and domains, fundamentally addressing decision-making problems. RL is a machine learning approach that involves interaction with the environment, mapping states to actions through a system of rewards and penalties [18]. This method primarily relies on an agent [19] collecting demand information from the environment in the current state, deriving rewards or penalties from the outcome of information mapping, driving the agent to maximize rewarded actions and transition to the next state. This iterative interaction generates a rational behavioral strategy, constituting the essence of self-learning for the agent through continuous updates driven by state–action relationships. In RL, the interaction between the agent and the environment is often conceptualized as a Markov decision process (MDP). Within this framework, the agent’s decision-making process in uncertain environmental conditions can be represented by a four-tuple model {*S*, *A*, *P*, *R*}. Here, *S*, *A*, and *R* correspond to the fundamental elements of RL: states, actions, and rewards, denoting the set of possible states, the set of all feasible actions based on states, and the reward function for state transitions, respectively. *P* reflects the state transition probability of the agent, representing the probability function of the agent taking different actions in different states. ML technologies have been extensively researched due to their adaptive learning capabilities, finding wide applications in autonomous agents and unmanned systems [20,21,22]. RL, as a branch of ML, optimizes operational strategies based on the interaction between agents and the environment. Particularly, in complex environments such as UAV path planning, RL demonstrates its robust learning capabilities [23,24].

The Q-learning [25] approach for path planning has been known as the next frontier in the field of UAV navigation and is noteworthy for not requiring the prior existence of rules or knowledge about the environment [26]. This cascade is the responsible element for the empowerment of the UAV, which behaves as an autonomous agent, able to make better trajectory decisions based on the rewards it accumulates from its actions within the mission field. The principal task for the UAV should be to create a route that fully encompasses the mentioned rewards, while at the same time safely going around obstacles, so that its target is reached. The q-table is the policy that is used to achieve this objective, where all the anticipated rewards to be had for each state–action pairing are carefully recorded. Such a table allows the UAV to select the actions that will receive the highest expected reward. With the UAV interacting and receiving feedback from its environment, the q-table will evolve and adapt to provide data on the actions with the greatest yields. The states in this context are the UAV’s precise positions and directions in which it is pointing. Reward metrics are changed separately, based on the UAV’s distance to its target and its interaction with obstacles. A greater reward is allocated for closer proximity to the goal, and a lesser reward for encounters with obstacles. However, both advantages and disadvantages can be encountered during the use of Q-learning in UAV path planning due to the high dimensionality of the space state–action, which leads to the slower performance of algorithms and less practical application.

To cope with the task of path planning, Q-learning for a group of robots was proposed, based on the results of [27], where Particle Swarm Optimization (PSO) was used. The effectiveness of this method is based on the prior assessment of the available routes within unknown environments. Another study [28] brings forward a Q-learning offline method that does not rely on the traditional dynamic programming or geometry-based methods; rather, it uses metrics that determine rewards by considering path length, safety, and energy. Another study [29] demonstrates a deterministic Q-learning model for mobile robots, which draws on known distances between states and goals and allows for planning the process in a more efficient way, thereby reducing the overall complexity of the planning process compared to alternative methods. An enhanced Q-learning algorithm, aimed at UAV path planning within hostile, unknown settings, was put forth in [30]. This work introduces innovative q-function initialization and action selection strategies to boost efficiency. Meanwhile, Ref. [31] explores a hybrid approach that integrates A-Star with Q-learning, evaluating UAV path planning through both local dynamic and global static lenses. The Adaptive Randomized Exploration (ARE) technique, presented in [32], addresses UAV path planning tasks, and a related method employing q-tilt for navigating uncertain dynamic contexts appears in [33,34]. In reference [35], Q-learning with adaptive stochastic exploration was employed to achieve UAV navigation and obstacle avoidance, though this approach is limited by action space and sample dimensionality. To address UAVs’ challenges in acquiring environmental data, reference [36] introduced a guided reinforcement Q-learning approach based on signal strength rewards, with simulations confirming the method’s advantages. However, standard RL techniques are generally suited only for simple discrete-state scenarios, struggling with high-dimensional inputs and the issue of dimensionality expansion. Reference [37] incorporated a Long Short-Term Memory (LSTM) network within a proximal policy optimization algorithm to propose a path planning approach, yet its scope is constrained by an environment limited to short walls. Reference [38] suggested an enhanced Deep Q-Network path planning method that addresses the Q-network’s overestimation issue. While path length and convergence speed comparisons highlight this algorithm’s strengths, it still falls short of fully optimizing the path.

Consequently, current research focuses on designing the shortest possible path to ensure that the UAV reaches its target while minimizing energy consumption. Although extensive studies have explored path planning in precision agriculture, research on the presence of dynamic obstacles remains insufficient. In complex orchard environments, dynamic obstacles such as birds introduce additional constraints to path planning algorithms, increasing the complexity of algorithm design and further complicating UAV path planning in precision agriculture.

In this study, we tackle the aforementioned challenges by proposing a path planning algorithm for precision agriculture that optimizes UAV energy efficiency while enabling obstacle avoidance. We conduct comprehensive research on obstacle avoidance and path planning tasks. The specific contributions of this paper are as follows:

1. The introduction of an improved Q-learning algorithm for UAV path planning that introduces SDP to the learning process, by which the UAV learns the location of the target point in the environment, thus reducing the learning time and the distance the UAV has to fly;

2. The introduction of an RMSP method with adaptive learning rate adjustment, allowing drones to modify their learning rates based on historical gradient data, thereby avoiding the problem of the overestimation or underestimation of learning rates, accelerating exploration convergence, and reducing drone energy consumption;

3. The introduction of deep neural networks into the improved Q-learning algorithm to solve the problem of dimensionality disaster and high computational complexity caused by the large state and action space in 3D environments.

The content of this article is organized as follows: Section 2 introduces the materials and methods; Section 3 describes the experiment and results; Section 4 is the discussion; and Section 5 summarizes the conclusions of this study.

## 2. Materials and Methods

### 2.1. 2D Path Planning

#### 2.1.1. Problem Modeling

(a) Problem Description

Q-learning-based UAV path planning aims to find the optimal collision-free path from the start to the end point to save energy. The UAV acts as an intelligent agent, updating the Q-table through repeated trials, selecting actions in different states, and learning from feedback. This process enables the algorithm to gradually find the optimal strategy [39] and ultimately allows the UAV to efficiently plan the shortest path in an unknown environment.

(b) Environment Modeling

Path planning is a fundamental element of both the safety and efficiency of UAV operations in areas where obstacles are present. The planning algorithm that does this integration puts together a number of critical factors like the geometrical dimensions and shapes of the obstacles as well as the UAV’s operational constraints and strategic objectives. Thus, it helps in finding routes which are not only safe but also well planned. In-depth knowledge of the surroundings is the main building block of an effective path plan. The accurate and detailed description of every obstacle in the operational environment is the key factor for the optimization of obstacle avoidance techniques and their effectiveness. The identification and differentiation of the categories of obstacles is a critical factor in the effective deployment of these methods. For example, at lower altitudes, UAVs are forced to penetrate rugged terrain and dense vegetation, which are major causes of collision. To address the challenges of complex environments, the path planning system employs geometric models to simplify obstacle representation, thereby reducing computational demands. Various shapes and sizes are used to represent obstacles like flying animals, trees, and fields, facilitating easier navigation.

**Figure 2:**
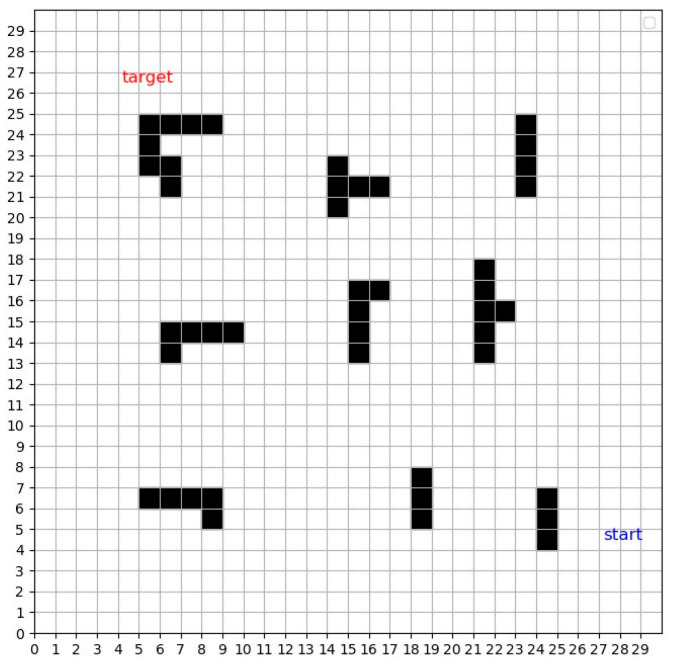
30 × 30 simulated grid obstacle environment.

The environmental setup of this study is shown in Figure 2 below, where a 30 × 30 grid is used, and each cell has a size of 20 pixels. The UAV’s position at the start is marked by a blue icon, while its intended target location is indicated by a red icon. Static and dynamic obstacles of different shapes and sizes in the environment are represented by black and red squares. The primary goal of the UAV is to navigate from the starting point to the target effectively, optimizing route efficiency to reduce both operational costs and time, while safely avoiding obstacles and minimizing the risk of collisions.

(c) Discrete action set

Using a gridded environment space is a common method in Q-learning UAV path planning [40], where a continuous space is divided into discrete grids to represent the UAV’s position, orientation, and other states [41]. The UAV’s flight environment can be abstracted as a tuple *(E, O)*, where *o ∈ O* denotes the obstacle grid and *e ∈ E* denotes the gap grid. The UAV’s action determines whether its position is an obstacle grid or a gap grid, as shown in Equation (1):

(1) $$\begin{array}{c} L\left(s_{t},a_{t}\right)=l_{t},l_{t}\in \left\{\begin{array}{c} E,l_{t}=e\\ O,l_{t}=o \end{array}\right. \end{array}$$

**Figure 3:**
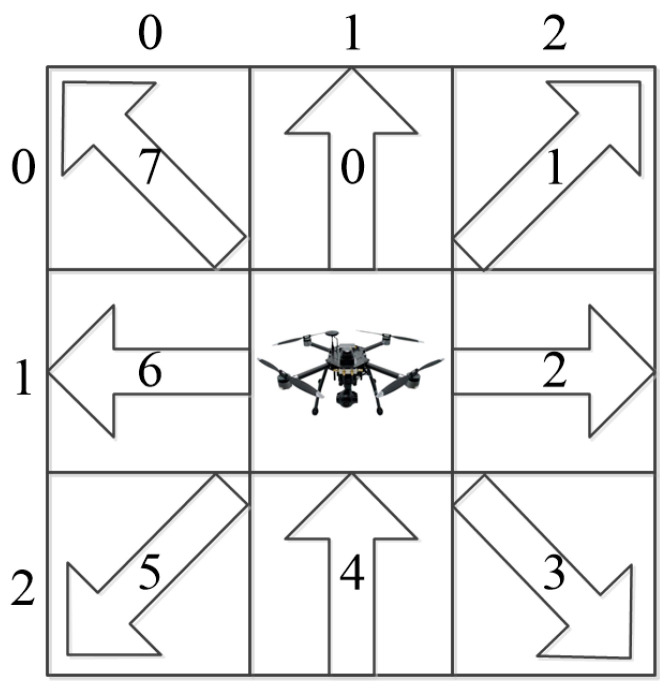
Discrete action set.

We can assume that the UAV is capable of moving to any of the four adjacent grid cells. Therefore, we define eight basic discrete movements: upward, upward-right, rightward, downward-right, downward-left, leftward, and upward-left. Each movement corresponds to a unit length, and these movements are numbered from 0 to 7, as illustrated in Figure 3.

#### 2.1.2. Q-Learning Algorithm

**Figure 4:**
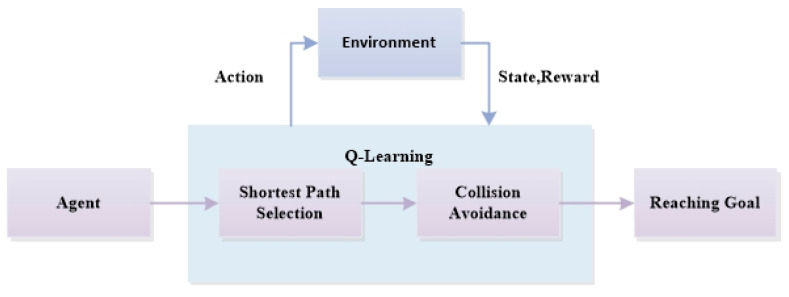
UAV trajectory planning and collision avoidance training framework.

The operational framework of this path planning and avoidance collision system is depicted systematically in Figure 4. The approach is structured around two pivotal components: the first is about the shortest path selection mechanism, where the UAV evaluates and chooses its next move based on the least distance strategy from its current location; the second one is about the obstacle avoidance protocol, which guides the UAV to change its flight path proactively to steer away from possible obstacles. These capabilities are developed to endow the UAV with the attributes to move through complex terrains efficiently and at low cost. This study also utilizes the RMSP method to dynamically adjust the learning rate based on gradient changes, enhancing learning speed and improving the efficiency of path planning.

**Figure 5:**
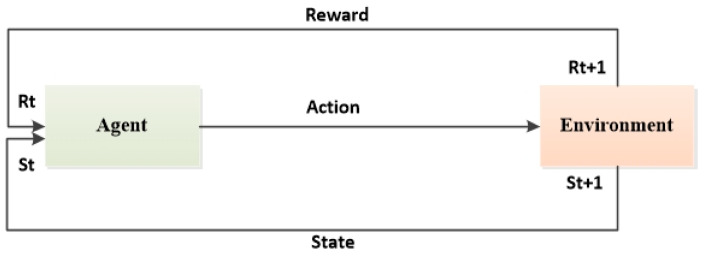
Structure of Q-learning algorithm.

Q-learning, an RL algorithm based on temporal difference, is used in order to define the q-table that stores the state–action values. This clear and precise rule-free method does not require complete environment knowledge, as a grid graph is used to travel and solve problems. The purpose of the algorithm is to get the agent to perform the task in a manner that leads to the maximum reward based on the interactions of the agent within its environment, as shown in Figure 5.

Q-learning’s architecture comprises five fundamental elements: the agent, the environment, states, actions, and the definition of rewards. Its environment is navigated by a framework that is based on punishments and rewards. Actions are performed by the agent, who considers the rewards associated with each of them and then carries them out. The agent interacts with the environment through a number of states, each of which is associated with a specific reward, and the agent makes its choice from this pool. Each action taken is associated with a reward and results in a change of system states. The choice of action for the agent is influenced by its strategy, and the rewards depend on the states. This loop can continue until the agent reaches the terminal state. During this procedure, the agent is learning from the states and, therefore, choosing actions to reach a higher reward. In reinforcement learning, an offline policy refers to a strategy that utilizes pre-collected data for training without the need for real-time interaction with the environment, while a solver is a policy or value function used to learn from previously collected data. Q-learning has the advantage of applying the TD method to solve offline learning problems and using the Bellman equation to formulate optimal strategies for Markov decision processes. As a result, Q-learning has been the subject of thorough research and application by numerous researchers and practitioners, demonstrating its clear, effective teaching of best strategies in complex environments.

RL is fundamentally concerned with solving the sequential decision-making challenge characterized by an MDP [42]. During the intelligent agent’s learning process, the Bellman equation is constructed using a value function and solved through Q-learning. In probabilistic scenarios, the state transitions and rewards resulting from actions are stochastic. To determine future actions in a given state, specific rules, referred to as policies, must be followed. This approach is central to reinforcement learning algorithms and is typically modeled using an MDP. In RL, the environment is evaluated based on several parameters. The state represents the agent’s observation of the environment, while actions correspond to the set of behaviors the agent can execute in a specific state. Rewards are used to guide learning numerically through the state transition probability, which describes how the agent moves between states [43]. Discount factors capture the diminishing value of future rewards over time, and policies define the rules that guide the agent’s action choices in any given state. A policy is essentially a mapping from states to actions, determining the likelihood of the agent selecting a specific action in a particular state [44], as shown in Formula (2):

(2) $$\mathrm{Pr}(\lambda |\gamma )=P[\Lambda_{t}=\lambda |\Gamma_{t}=\gamma ]$$

Here, *Pr* represents the probability of the agent choosing a specific action λ in a specific state γ.

In the domain of reinforcement learning, the value function is pivotal in assessing which policies will provide the most advantageous outcomes in future actions. This function estimates the aggregate of all anticipated rewards that are expected to accumulate by following a specific policy over time. In the Q-learning framework, value functions are categorized into two primary types: state value functions and action value functions. Each plays a critical role in the algorithm’s ability to learn and make decisions. The state value function specifically measures the expected return starting from a particular state and following a policy thereafter. The method to calculate this state value function is outlined as follows:

(3) $$v_{p}(\gamma )=E_{p}[R_{t+1}+\delta v_{p}(\Gamma_{t+1})|\Gamma_{t}=\gamma ]$$

In this model, *p* denotes the policy selected by the agent, *R_t+1_* represents the reward expected at the next time step, and *γ* symbolizes the discount factor. Equation (3) defines a state value function, which calculates the aggregate rewards expected from future states based on the chosen policy. It represents the total reward obtained by selecting a certain state, γ. On the other hand, the action value function describes the expected return the agent will receive by taking a specific action, λ, in state γ. The function that assesses the viability of selecting particular actions under policy p is known as the action value function, commonly referred to as the Q function. The primary objective of the Q-learning algorithm is to refine this function to achieve the optimal action value, designated as Q*. The agent is instructed to learn various paths by the following formula (4):

(4) $$Q*(\gamma ,\lambda )=E_{p}[R_{t+1}+\delta \underset{\lambda \in \Lambda }{max}Q*(\Gamma_{t+1},\lambda^{\prime })|\Gamma_{t}=\gamma ,\Lambda_{t}=\lambda ]$$

Among them, $\lambda^{\prime }$ represents the next action, and $Q^{*}(\gamma ,\lambda )$ can be approximated as $\tilde{Q}(\gamma ,\lambda )$ at time *t*. The expected state at the next moment is denoted as $\Gamma_{t+1}$. The agent executes an action, $\Lambda_{t}$, from the current state, $\Gamma_{t}$, to transition to the subsequent state $\Gamma_{t+1}$ and receives the corresponding reward, $R_{t}$, from that environment. The dynamic known as the agent–environment interaction, or agent–environment loop, describes the process where an agent continuously interacts with its environment. The relationship between the Q function, which assesses the feasibility of specific actions within a policy, and the value function, which aggregates potential rewards, is illustrated below.

(5) $$v_{p}(\gamma )=\sum_{\lambda \in \Lambda }P(\lambda |\gamma )Q*(\gamma ,\lambda )$$

The value function is derived by summing the respective Q functions for each action with the value assigned by the policy. The value function and Q function are defined through the Bellman equation, which clarifies the relationship between the value functions of the current state and future states. The Q-learning algorithm is built around two main components. The initial phase involves collecting training data under a specific predetermined policy, which can be adjusted according to the specific goals of the problem at hand. The agent interacts with the environment by generating estimated trajectories influenced by these policies. These trajectories can subsequently be segmented into four distinct parts: $\Gamma_{t},\Lambda_{t},R_{t},\Gamma_{t+1}$. This approach is referred to as the behavioral strategy, which is implemented to manage agent actions within their environment. One of the most prevalent forms of behavioral strategies is the ϵ-Greedy strategy, which is characterized as follows:

(6) $$\left\{\begin{array}{c} \underset{\lambda }{\arg\;\max\;}\tilde{Q}(\Gamma_{t},\lambda ),\;1-\varepsilon \\ Uniform,\;\;\;\;\varepsilon \end{array}\right.$$

In this research, the ϵ-Greedy algorithm is utilized to determine the actions taken by agents within various states. The agent executes a chosen action and consequently receives a reward, prompting a transition to another state. This Q-learning approach incorporates the greedy strategy, systematically selecting actions in each state to optimize rewards. An important component of Q-learning discussed here is experience replay, which utilizes memory replay technology to store the agent’s experiences at each step. From this memory, a randomly chosen set of data, ${(\Gamma }_{t},\Lambda_{t},R_{t},\Gamma_{t+1})$, is extracted.

The variables $\left(\Gamma_{t},\Lambda_{t},\Gamma_{t+1},{\tilde{Q}}_{now},{\tilde{Q}}_{new}\right)$ record the status, actions, rewards, and the status of the result between the influencer and the environment. The estimated *q* values, ${\tilde{Q}}_{now}$ and ${\tilde{Q}}_{new}$, are also included in $\left(\Gamma_{t},\Lambda_{t},\Gamma_{t+1},{\tilde{Q}}_{now},{\tilde{Q}}_{new}\right)$, and the value of ${\tilde{Q}}_{now}$ at positions $\gamma_{j}$ and $\lambda_{j}$ is saved as ${\hat{q}}_{j}$. Then, the maximum state value of $\Gamma_{t+1}$ in ${\tilde{Q}}_{new}$ is calculated.

(7) $${\hat{q}}_{j+1}=\underset{\lambda }{\max}{\tilde{Q}}_{now}(\gamma_{j+1},\lambda )$$

The TD target is then calculated as follows:

(8) $${\hat{y}}_{j}=r_{j}+\gamma {\hat{q}}_{j+1}$$

The calculation result of TD error is as follows:

(9) $$\delta_{j}={\hat{q}}_{j}-{\hat{y}}_{j}$$

Based on this, the new estimate for function ${\tilde{Q}}_{new}$ of the action value in its current state is determined as follows:

(10) $${\tilde{Q}}_{new}(\gamma_{j},\lambda_{j})\leftarrow (1-\alpha ){\tilde{Q}}_{now}(\gamma_{j},\lambda_{j})+\alpha \delta_{j}$$

Among them, α and γ are hyperparameters representing learning rate and discount factor, respectively. The value of α is between 0 and 1.

#### 2.1.3. Improved Q-Learning

(a) Shortest distance priority

Energy conservation is crucial in UAV applications. To address this issue, minimizing the travel distance UAVs need to cover to reach their targets is crucial. An efficient path-planning approach can achieve this goal by reducing the unnecessary distance traveled by the UAV. According to the original Q-learning algorithm, we propose a shortest distance prioritization policy for Q-learning that first tries to find the shortest route as the primary goal and is different from the ϵ-greedy strategy that is currently used in more traditional models. By means of the new algorithm, the UAVs do not have to travel so much, as shown in Algorithm 1.

**Algorithm 1 **Our proposed Q-learning algorithm for the UAV path planning problem.**Input: **Source location, destination location, and solution space **Output: **Optimal path for UAV from source to destination 1: Initialize** **Q(γ,λ)←0(Γ states, Λ actions); 2: **for **each episode** do** 3:    set $\gamma_{t}\leftarrow \lambda$ random state from state set Γ; 4:    **while** $(\gamma_{t}\neq \mathrm{target})\;$**do** 5:      ** for** each $\lambda_{t}^{i}\in \Lambda$ where i∈[up,down,left,right]** do** 6: **         **Determine location** **$loc_{\lambda_{t}^{i}}$** **of agent by doing action $\lambda_{t}^{i}$ **7:          **Calculate distance** **$dist_{t}^{i}\in dist_{t}$** **from** **$loc_{\lambda_{t}^{i}}$** **to Target location. 8:          Choose $loc_{\lambda_{t}^{i}}$** **corresponds to smallest** **$dist_{t}^{i}$** **from $dist_{t}$ 9:         Choose $\lambda_{t}^{i}$ corresponds to $loc_{\lambda_{t}^{i}}$** **which makes the agent move closer to Target location 10:       **end** 11:       Perform action $\lambda_{t}^{i}$ and receive penalty or reward 12:      Update $Q(\gamma_{t},\lambda_{t})$ 13:   **end** 14: **end **

(b) Dynamic learning rate optimization adjustment

In Q-learning algorithms, dynamic learning rate tuning is crucial to improve performance. Traditional Q-learning uses a fixed learning rate, but dynamic environments may lead to its inadequacy.

RMSP dynamically adjusts the learning rate based on environmental changes by calculating the sliding average of the gradient squared [45]. The key concept is to integrate the gradient history information so that different parameters have different learning rates. The learning rate α is updated based on the rules of Equations (11) and (12).

(11) $$E[{ɡ}^{2}{]}_{t}=0.9E[{ɡ}^{2}{]}_{t-1}+0.1{ɡ}_{t}^{2}$$

(12) $$\alpha_{t+1}=\alpha_{t}-\frac{\alpha_{0}}{\sqrt{E[{ɡ}^{2}{]}_{t}+\lambda }}{ɡ}_{t}$$

where ${E[{ɡ}^{2}]}_{t}$ represents the expected value of the gradient squared, ɡ is the current gradient, $\alpha_{0}$ is the starting learning rate, and λ is a minor constant applied to avoid division by zero. To implement RMSP in Q-learning, the Q-table update process is modified accordingly. Specifically, the update rule for state $s_{t}$ and action *t* in the Q-table is represented in Equation (13):

(13) $$Q(s_{t},a_{t})=Q(s_{t},a_{t})+\alpha_{t}\frac{\alpha_{0}}{\sqrt{E[\nabla Q(s_{t},a_{t}{)}^{2}]+\lambda }}\delta$$

where *δ* represents the temporal difference (TD) error, and $E[{▽Q(s_{t},\alpha_{t})}^{2}]$ signifies the rolling average of the squared gradient. The detailed algorithmic flow is presented in Algorithm 2.

**Algorithm 2 **Dynamic Learning Rate Adjustment Algorithm.**Require:** Initial Q value, learning rate a decay coefficient for the moving average of squared gradients β, small constant λ **Ensure: **Optimized Q value 1: Initialize** **Q-values randomly or with prior knowledge 2: Initialize $E[\nabla Q^{2}]$ to a small positive value 3: **for **each episode** do** 4:    **for** each step in the episode **do** 5:    Choose an action α using the combined ε-greedy and Boltzmann strategy 6:    Take action α observe the next state $s^{\prime }$ and the reward r 7:    Calculate the TD error δ 8:    Update $E[\nabla Q^{2}]$ using the decay factor β 9:    Update Q-values using the RMSprop update rule 10:   Move to the next state s 11:    **end for** 12: **end for** 13: **return **Q-values

### 2.2. 3D Path Planning

#### 2.2.1. Deep Q-Learning Algorithm

In the previous subsection, we proposed the improved Q-learning algorithm. The limitation in 3D path planning mainly reflects the complexity of the scale of the state and action space, and the number of states and actions increases with the spatial dimension so that the Q-table needs to occupy a large amount of space and slow down the convergence of the algorithm to reduce the path planning effect. Therefore, on this basis, deep neural networks were used to calculate Q-values. They map the continuous state space to the Q-value space through deep neural networks, which not only solves the storage problem, but also efficiently handles complex state information.

**Figure 6:**
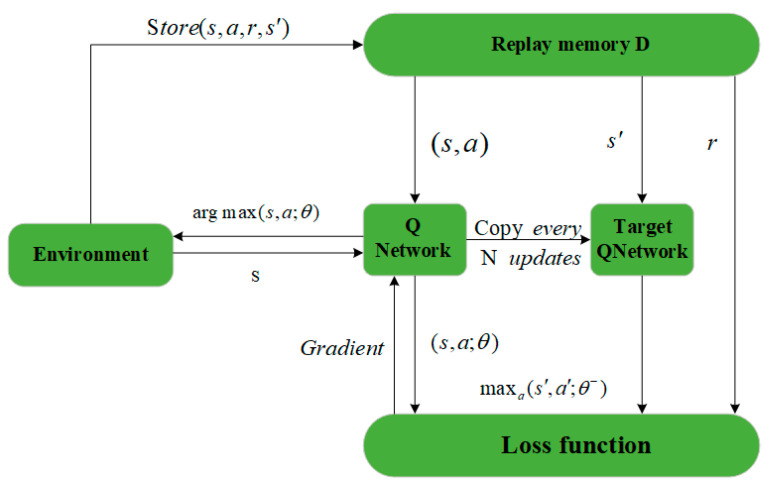
Deep Q-Learning algorithm structure.

The Deep Q-learning algorithm has two models with the same structure; one is the Q-network and the other is the target Q-network. The parameters of the target Q-network are kept for a period of time, the purpose of which is to avoid algorithmic divergence. The algorithm updates the values of the Q-network parameters to the target Q-network after a period of time, as shown in Figure 6.

The core of Deep Q-Learning is to compute the error between the outputs of the Q network and the target Q network in order to update the Q approximation. It first initializes the Q network and the target Q network and builds an experience playback pool to store the interaction data of the intelligences. The UAV selects an action based on the Q network at each step and stores the state, action, reward, and new state after execution. The algorithm randomly samples data from the playback pool, estimates the maximum Q value of the next state using the target Q network, and combines the discount factor and instant reward to form the target Q value. Then, it calculates the error with the predicted value of the Q network and back-propagates to update the parameters. Through many iterations, Deep Q-Learning continuously optimizes the Q value to achieve a better strategy, as shown in Algorithm 3.

**Algorithm 3 **Deep Q-Learning with Experience Replay.1: Initialize replay memory D to capacity N; 2: Initialize action-value function Q with random weights 3: **for** episodes = 1, M** do** Initialize sequence $s_{1}=\{x_{1}\}$ and preprocessed sequenced $\varphi_{1}=\varphi (s_{1})$ 4:   **for** t = 1,T **do** 5:   With probability ε select a random action select a random action $\alpha_{t}$ 6:   otherwise select $\alpha_{t}=\max_{a}Q^{*}(\varphi (s_{t}),\alpha ,\theta )$ 7:   Execute action $\alpha_{t}$ in emulator and observe reward $r_{t}$ and image $x_{t+1}$ 7:   Set $s_{t+1}=s_{t},\alpha_{t},x_{t+1}$ and preprocess $\varphi_{t+1}=\varphi (s_{t+1})$ 8:   Store transition $\left(\varphi_{t},\alpha_{t},r_{t},\varphi_{t+1}\right)$ in D 9:   Sample random minibatch of transitions $\left(\varphi_{j},\alpha_{j},r_{j},\varphi_{j+1}\right)$ from D 9:   Set $y_{j}=$ using the RMSprop update rule 10:   Perform a gradient descent step on $(y_{j}-Q(\varphi_{j},a_{j};\theta ){)}^{2}$ 11:    **end for** 12: **end for**

The purpose of experience replay is to utilize the supervised learning property of deep neural networks, which is that the data need to be independently and identically distributed. However, Q-learning produces samples that are correlated with neighboring data. Experience replay improves training stability and efficiency by storing the interaction data of the intelligences and randomly sampling them for training, breaking the correlations between the data. The following equations are for the gradient descent method and for the loss function:

(14) $$g_{1}=(r+r\max_{a^{\prime }}Q(s^{\prime },a^{\prime };\theta_{1}^{\prime })-Q(s,a,\theta_{1})){▽}_{\theta }Q(s,a;\theta )$$

(15) $$L_{1}(\theta_{1})=E[(r+r\max_{a^{\prime }}Q(s^{\prime },a^{\prime };\theta_{1}^{\prime })-Q(s,a,\theta_{1}){)}^{2}]$$

The stochastic gradient descent method is used to optimize the weights and minimize the loss function so as to progressively approximate the optimal Q-function that satisfies Bellman’s equation. When the loss function tends to zero, the approximation function can be considered to have reached the optimal solution.

#### 2.2.2. AirSim Simulation Environment

**Figure 7:**
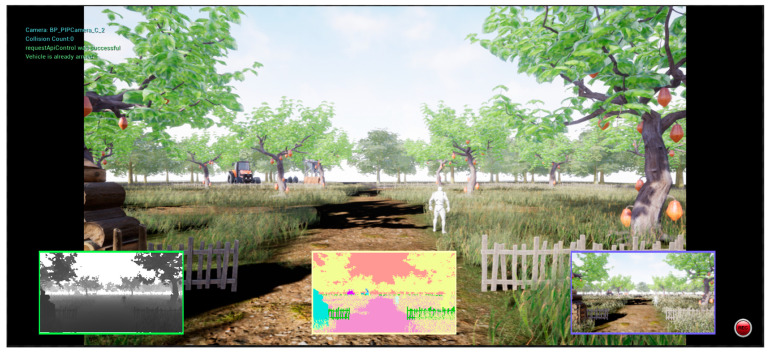
Screen shot of the AirSim environment.

AirSim is a high-fidelity, open-source simulation platform developed by Microsoft and designed for UAV and self-driving car R&D. Built on the Unreal Engine and Unity engine, AirSim provides realistic physical environments and visual effects. When using reinforcement learning for UAV obstacle avoidance training, the simulation provides a safe, low-cost trial-and-error environment that supports rapid iteration and complex scene simulation to efficiently optimize the algorithms without causing worry about flight risks and costs. AirSim’s environment is shown in Figure 7.

**Figure 8:**
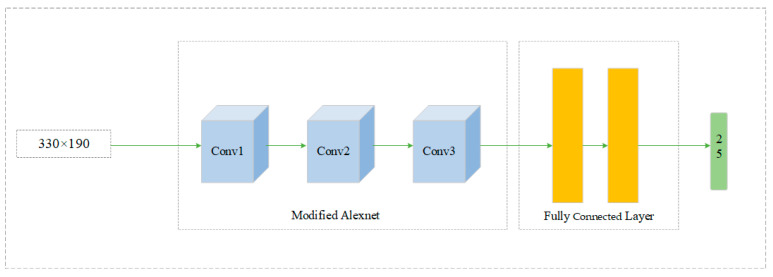
Neural network architecture.

In this study, there is an RGB camera and depth camera in front of the UAV in Airsim. For the UAV, the RGB image obtains the current features, and the depth image obtains information about the distance to the surrounding obstacles and is rewarded for the current action. We used the back-propagation method to train the deep neural network using the error between the target value and the current value. Afterward, we output the best predicted Q-value used for action selection. Figure 8 shows the neural network architecture. The image uses 330 × 190 as input; it performs feature extraction through a convolutional network layer, then classification through a fully connected layer, and finally outputs 25 Q-values for each action selection.

**Figure 9:**
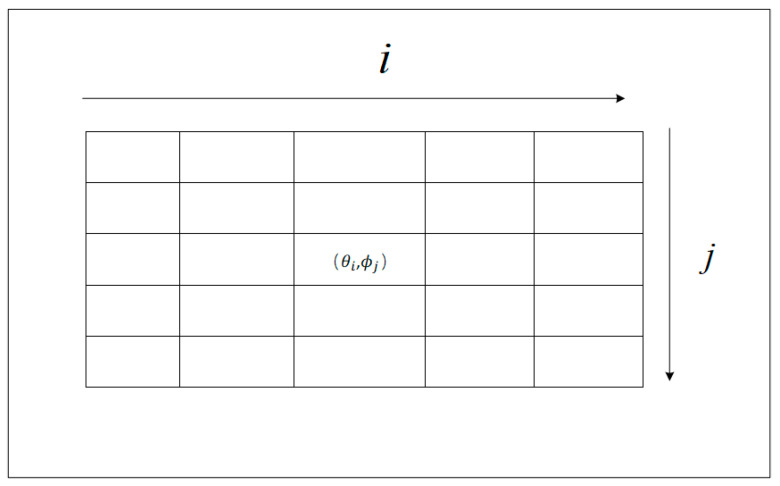
The 5 × 5 action space.

The operation space is a 5 × 5 size, as shown in Figure 9. There are 25 operation options; the flight movements of the UAV are calculated by the angle equations, and then we output the pitch and yaw control commands to the UAV and indicate the horizontal and vertical movement angles. These angle equations are calculated as follows:

(16) $$\theta_{i}=(\frac{FOV}{N^{2}}\times \frac{i-(N^{2}-1)}{2})$$

(17) $$\phi_{j}=(\frac{FOV_{v}}{N^{2}}\times \frac{i-(N^{2}-1)}{2})$$

where i and $j\in \{0,1,......,\frac{N^{2}-1}{2}\}$ are the locations, as shown in Figure 9. The size of the action space is N × M. $FOV_{h}$ and $FOV_{v}$ provide information on the horizontal and vertical attitudes, respectively, taken from the UAV.

## 3. Results

### 3.1. 2D Simulation Experiments

#### 3.1.1. Experimental Environment and Setup

The experiment of this study was conducted in a simulation environment using Ubuntu 18.04 and Anaconda3. The experimental program is based on Python 3.8. All experiments were conducted on NVIDIA GeForce RTX 4060 GPU and Intel Core i9-11800H CPU. The parameters of the comparison algorithm are shown in Table 1.

#### 3.1.2. Evaluation Indicators

Shortest Distance: This metric assesses whether the algorithm can identify the shortest path from the starting point to the endpoint. The shortest path length is often a key indicator of the efficiency of path planning algorithms, particularly in terms of time, resources, and cost savings.

Steps: The number of actions executed by the UAV from the initial state to the termination state within an episode represents the step count. This metric reflects the intelligence’s exploration and learning efficiency within the environment. Typically, the step count is higher at the outset, gradually decreases as the strategy is optimized, and may increase in complex or dynamic environments. It serves as a critical indicator of both the algorithm’s convergence speed and the environment’s complexity.

Reward: The reward value is numerical feedback provided by the environment after the UAV executes an action, used to assess the quality of that action. It guides the intelligent agent in learning optimal strategies through positive, negative, or neutral feedback, influencing both immediate behavior and playing a critical role in long-term strategy development. This mechanism is central to optimizing decision-making in reinforcement learning.

Learning Rate: The learning rate reflects the degree to which the agent’s experience gained during exploration contributes to updating the new Q-values.

#### 3.1.3. Comparison of Shortest Distances

**Figure 10:**
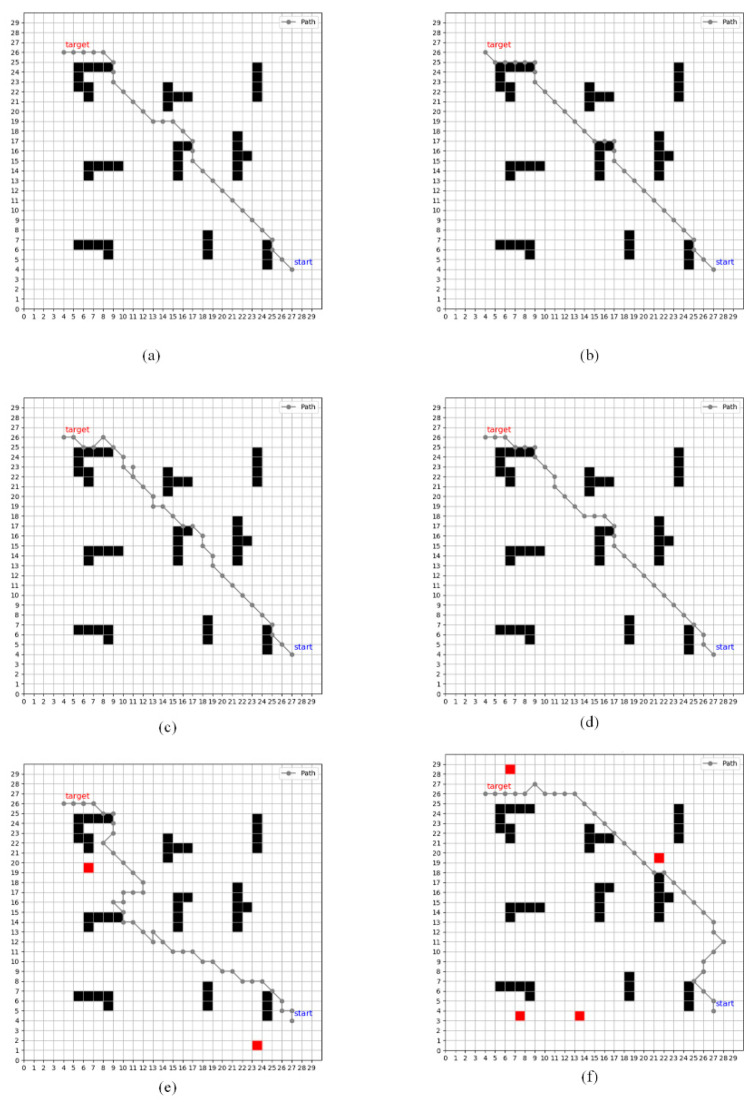
Performance of various unmanned aerial vehicle path planning algorithms in the presence of obstacles. (**a**) A-star. (**b**) Dijkstra. (**c**) Original Q-learning. (**d**) Proposed Q-learning. (**e**) Proposed Q-learning in the presence of two dynamic obstacles. (**f**) Proposed Q-learning in the presence of four dynamic obstacles.

To assess the performance of our newly developed method, we performed a comparative analysis with established path-planning techniques, focusing on execution time and path efficiency. Initially, we examined the A-star algorithm, a well-regarded and frequently used method in path planning that combines graph traversal with heuristic techniques for rapid and reliable route determination. The paths generated by the A-star algorithm within our test environment are depicted in Figure 10a. We also compared our method with the Dijkstra algorithm, a renowned technique for finding the shortest paths in a weighted graph from a single source node to all other nodes. It calculates the minimal cumulative distance for all nodes from the source, as demonstrated in Figure 10b. Further comparisons were made with the original Q-learning algorithm. Figure 10c,d demonstrate the effect of the Q-learning and improved Q-learning algorithm in static environments, and it is clearly shown that the improved Q-learning paths are shorter in distance. We evaluated performance in the presence of multiple moving obstacles, and Figure 10e,f demonstrate the effect of the improved Q-learning algorithm in two and four dynamic obstacle environments, respectively. The distance traveled in dynamic environments is increased compared to static environments due to the introduction of dynamic obstacles, which cause the UAV to explore the environment in a complex manner.

Unlike the A-star and Dijkstra algorithms, the Q-learning algorithm does not rely on pre-existing environmental data, making it potentially more suitable for real-time UAV operations. The path lengths and computation times for these algorithms are presented in Table 2, where it is evident that while A-star and Dijkstra excel in speed and minimal path distance, their requirement for prior environmental knowledge limits their utility in certain UAV contexts. Our enhanced Q-learning algorithm, as shown in Figure 10d, effectively reduces the path distance required, aligning with the real-time operational needs of UAVs.

The comparative results, illustrating differences in path length and training time, are summarized in Table 2. This analysis demonstrates that our improved Q-learning algorithm not only optimizes the path length but also enhances adaptability to diverse operational environments without the need for pre-mapped data.

#### 3.1.4. Comparison of Steps

**Figure 11:**
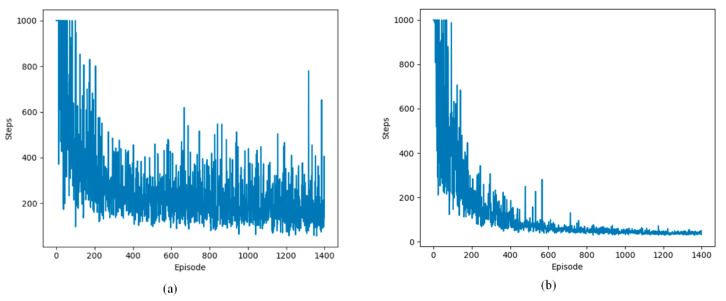
Changes in step count during training in a static environment. (**a**) steps of Q-learning algorithm; (**b**) steps of proposed Q-learning algorithm.

**Figure 12:**
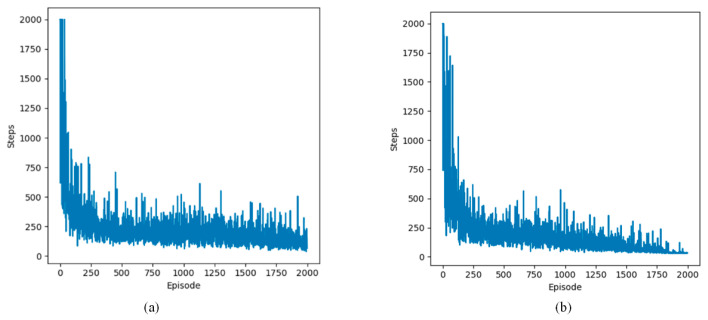
Changes in step count during training in two dynamic environments. (**a**) steps of Q-learning algorithm; (**b**) steps of proposed Q-learning algorithm.

**Figure 13:**
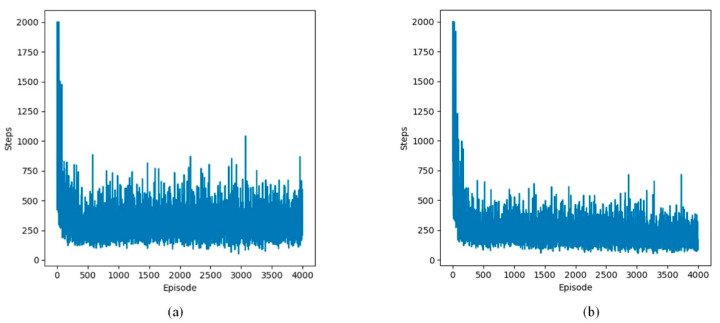
Changes in step count during training in four dynamic environments. (**a**) steps of Q-learning algorithm; (**b**) steps of proposed Q-learning algorithm.

This section examines the change in the number of steps per episode in defined static and dynamic grid environments. The experimental results from static and dynamic environments are depicted in Figure 11, Figure 12 and Figure 13.

The following lines represent two Q-learning-based path planning algorithms. The experimental results show that the algorithms exhibit fluctuations initially and then decrease and stabilize in the three environments. The initial fluctuation stems from the subject’s limited understanding of the environment, leading to more exploratory maneuvers. As the number of arrivals at the terminal node increases, the UAV is able to recognize the shortest path faster due to accurately learning the environment information.

#### 3.1.5. Comparison of Reward

**Figure 14:**
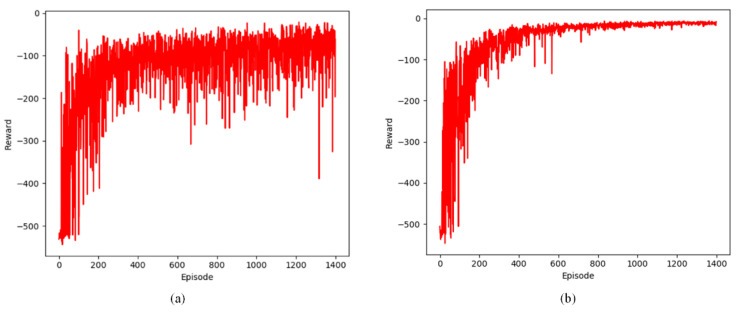
Changes in cumulative rewards during training in a static environment. (**a**) steps of Q-learning algorithm; (**b**) steps of proposed Q-learning algorithm.

**Figure 15:**
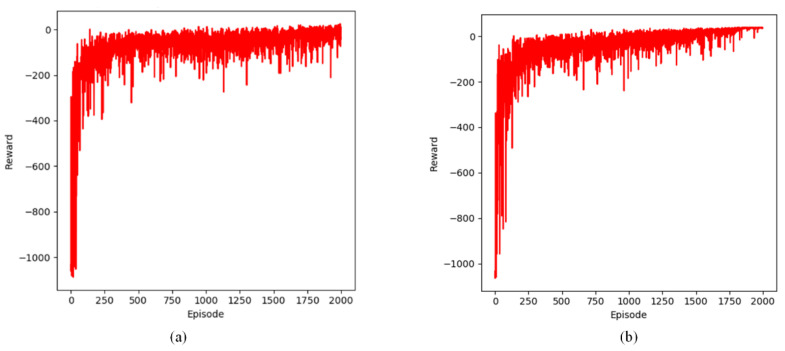
Changes in cumulative rewards during training in two dynamic obstacle environments. (**a**) steps of Q-learning algorithm; (**b**) steps of proposed Q-learning algorithm.

**Figure 16:**
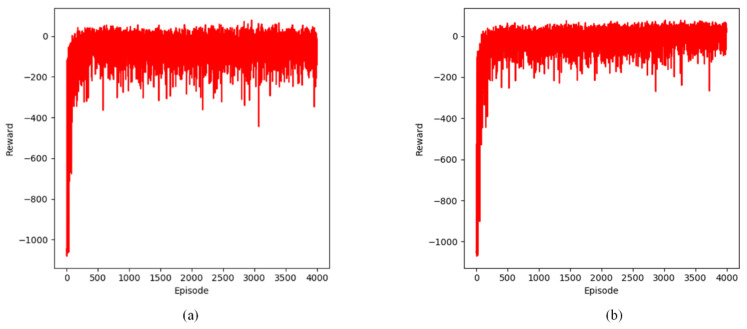
Changes in cumulative rewards during training in four dynamic obstacle environments. (**a**) steps of Q-learning algorithm; (**b**) steps of proposed Q-learning algorithm.

This section examines the variation in reward values across episodes within the defined static and dynamic grid environments. The experimental results for both static and dynamic environments are presented in Figure 14, Figure 15 and Figure 16.

The graphs show the performance of the Q-learning-based path planning algorithm in both static and dynamic environments. The experimental results show that the reward value per episode in the early exploration phase is negative and at a low level. As the agent’s steps gradually converge, the reward value also gradually converges to a higher level, indicating that the agent believes that the shortest path has been found.

Our improved Q-learning algorithm exhibits the quickest convergence rate and the highest reward asymptotic value. This can be ascribed to the use of a shortest distance strategy and a dynamic learning rate, which accelerates convergence by guiding the agent toward the endpoints while preventing it from revisiting the same grid within the current episode and minimizing negative rewards caused by collisions. In contrast, the primitive Q-learning algorithm employs a fixed learning rate, resulting in unguided agent movement and difficulties in learning environmental features, particularly in complex environments, which can lead to slower updates.

#### 3.1.6. Comparison of Learning Rates

The horizontal axis of the chart shows the number of episodes, and the vertical axis shows the learning rate when the UAV updates the Q-table. Distinct curves align to different obstacle densities. The improved Q-learning algorithm uses RMSP, featuring dynamic and adaptive learning rate adjustment, while the original Q-learning algorithm uses a constant learning rate set to 0.001. As the episode progresses, the improved Q-learning algorithm adjusts its historical-based learning rate about the gradient to adapt to changing and unfamiliar environments.

**Figure 17:**
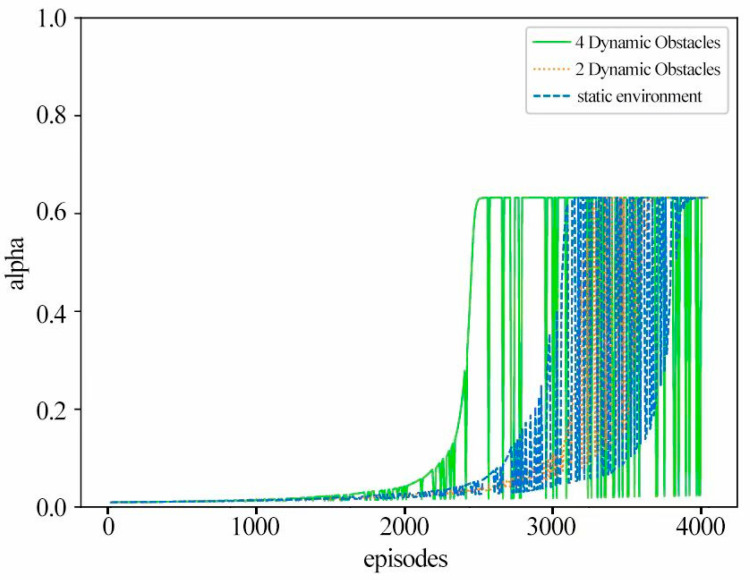
Changes in learning rates.

Figure 17 shows the change in learning rate. In scenarios with three different obstacles, the learning rate follows a consistent trend: it remains stable, around 0.001 for the first 2000 episodes, then gradually increases, accompanied by fluctuations. This model stems from the simple state transitions that the UAV encounters initially, where the gradient information is consistent and the learning rate fluctuates less. As training progresses and the UAV explores more complex states, gradient changes intensify, leading to learning rate adjustments. In addition, the diversity of reward signals also triggers learning rate fluctuations, and state-specific reward signals may prompt rapid gradient changes, thus affecting the dynamic adaptation of the learning rate.

### 3.2. 3D Simulation Environment

#### 3.2.1. Experimental Setup

Path planning for a quadcopter UAV in an orchard environment is demonstrated using the Microsoft AirSim 1.8.1 simulation software environment. The simulation experiment was flown with a photo input size of 330 × 190. The number of training iterations was 120,000, the batch size was set to 32, the discount factor to 0.95, and the initial learning rate to 0.000008. The training parameters are shown in Table 3.

#### 3.2.2. UAV Orchard Obstacle Avoidance

**Figure 18:**
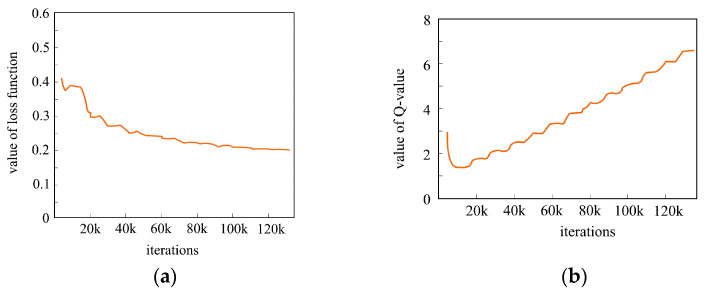
Training process diagram. (**a**) represents the loss function plot and (**b**) represents the maximum reward value.

During the training process, the loss function starts to diverge after more than 120,000 training sessions, resulting in a decrease in the model’s training effectiveness. After many iterations, the Q increases to achieve the overall highest reward, as shown in Figure 18.

**Figure 19:**
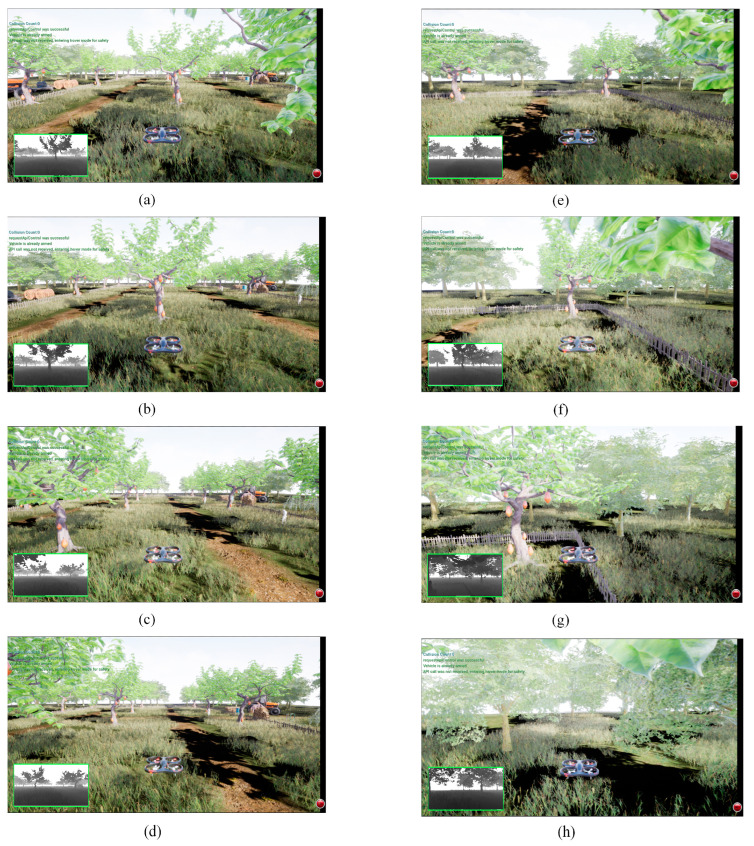
The obstacle avoidance process of UAV. (**a**–**d**) represents the UAV avoiding obstacles; (**e**–**h**) represents the UAV crossing an obstacle.

We conducted two tests in an orchard simulation environment. In the first example, the UAV sees a fruit tree from a distance, as shown in Figure 19a, and chooses to fly to the right based on the trained Deep Q-Learning algorithm. Figure 19b shows the UAV beginning to change direction and yawing to the right. Figure 19c shows the UAV moving to the right of the fruit tree, and eventually the UAV successfully navigates around the obstacle, as shown in Figure 19d. The UAV was able to navigate to the right of the fruit tree. On another occasion, there were two dense trees in front of the UAV. From the depth map (Figure 19e), it can be seen that there was a navigable space between the two trees. As the UAV gradually approached the trees (Figure 19f), the gap between the two was wide enough for the UAV to pass through. During the penetration process, the UAV did not fly in a completely straight line, but changed its direction slightly (Figure 19g), after which it gradually adapted to the open area. Eventually, the UAV successfully passed through the obstacle, as shown in Figure 19h.

## 4. Discussion

Currently, many scholars are focusing on path planning research in precision agriculture. For instance, Huang et al. [46] proposed an autonomous task assignment and decision-making method for the overlay path planning of a multi-collaborative quadcopter, aimed at UAV-based medication spraying in agricultural scenarios. Castro et al. [47] introduced an online adaptive path planning solution, combining RRT and DRL algorithms. This approach was employed to generate and control autonomous UAV trajectories during flytrap inspection missions in olive cultivation. Additionally, Goodrich et al. [48] developed a novel sequential gap reduction algorithm to determine sensor placements in agricultural fields, minimizing sensor overlap and optimizing sensor locations across four different types of agricultural fields. Collectively, these studies have made significant contributions to the advancement of precision agriculture.

Q-learning enables the agent to learn the optimal policy through interaction with the environment, without requiring prior knowledge of the environment model, and is well suited for handling stochasticity and achieving rapid convergence. Thus, the Q-learning algorithm was ultimately chosen. A challenge lies in efficiently planning the most optimal path while learning the environment. To address this issue, De et al. [49] proposed an offline Q-learning approach for path planning, avoiding dynamic programming or geometry-based methods. This approach focuses on path length, security, and energy consumption as key factors in determining the reward.

However, all of the aforementioned methods exhibit various issues and fail to consider the presence of dynamic obstacles within the UAV’s operational environment. Therefore, the constraints on path planning algorithms become more complex as the number of obstacles in the environment increases, leading to greater time complexity in overall path planning—especially when dynamic obstacles are present, meaning their positions can change over time. To address this, a shortest-distance-first strategy is introduced during the learning process, which assigns higher priority to actions that move the UAV closer to the target. This approach reduces the distance the UAV travels to the target in each episode, minimizes blind search during exploration, enhances the algorithm’s learning efficiency, and shortens the UAV’s path. In traditional Q-learning algorithms, the learning rate is typically fixed, but in real-world scenarios, environmental dynamics can render a fixed learning rate inadequate. Thus, the RMSP method is employed, dynamically adjusting the learning rate based on gradient changes, allowing the algorithm to adapt to dynamic and unfamiliar environments, thereby enhancing the efficiency of path planning.

However, a significant challenge when applying Q-learning to path planning is the large state–action space, which can slow down the algorithm and render it impractical. In addition, traditional Q-learning methods rely on lookup tables to store the Q-values of each state–action pair, which performs well in low dimensional state spaces but cannot cope with the high-dimensional, continuous, and complex state spaces in real 3D scenes.

Therefore, in order to enable the algorithm to be implemented in real precision agriculture scenarios, we used an algorithm called Deep Q-Learning, which introduces deep neural networks into the Q-learning algorithm. Deep Q-Learning can learn optimal strategies for high-dimensional state spaces more efficiently by better estimating Q values in complex scenes. The state space complexity in 3D scenes is much higher than that in traditional 2D or low dimensional scenes, so Deep Q-Learning can better handle these complexities and high dimensions. In addition, after conducting 3D simulation experiments, we introduced deep neural networks into our improved Q-learning algorithm to achieve collision-free path planning for unmanned aerial vehicles, and there were no conflicts between the various improvements. Consequently, this approach is anticipated to generate a feasible, stable, safe, and optimal trajectory for UAVs tasked with data collection in orange orchards, supporting precision agriculture.

## 5. Conclusions

The algorithmic enhancement in this paper aims to overcome the slow convergence and inefficiency of traditional algorithms used for localized path planning while also slightly reducing the distance the UAV must travel to reach the target and saving energy. The performance of the proposed method was evaluated through 2D simulation experiments, with metrics such as shortest distance, reward, steps, and learning rate, and then was compared with methods including A-star, Dijkstra, and Q-learning. The results demonstrate that the proposed method achieves the minimum total distance traveled by the UAV and provides superior obstacle avoidance compared to these alternatives. In the real world, training UAVs using Q-learning methods will be expensive in terms of cost consumption. Therefore, we chose to conduct research in a 3D virtual environment and introduced deep neural networks into our proposed method to better adapt the algorithm to the 3D environment. The simulation results show that the algorithm can achieve complex terrain adaptation, dynamic obstacle avoidance, and distance optimization in the 3D environment of orchards. This indicates that the algorithm can be efficiently and reliably implemented in precision agriculture.

In future work, we plan to further expand the algorithm by optimizing path selection through energy consumption weights or energy constraints to ensure effective obstacle avoidance in three-dimensional environments while minimizing UAV energy consumption. We will also attempt to deploy the improved algorithm on unmanned aerial vehicles for real flight verification.

## Figures and Tables

**Table 1:** Experimental parameters.

Algorithms	Parameters
A-star	the length of come from = 1000
Dijkstra	the length of come from = 1000
Q-learning	$\gamma \in \left[0.1,0.9\right],\varepsilon =0.9$
Improved Q-learning	$b=5000,\beta =0.3,\gamma \in \left[0.1,0.9\right],\varepsilon =0.9,$

**Table 2:** Comparison of the performance of various unmanned aerial vehicle path planning algorithms in terms of time and distance.

Algorithm	Training Time	Shortest Distance
A-star	0.0133 s	31
Dijkstra	0.0185 s	49
Original Q-learning	348.3186 s	70
Proposed Q-learning	332.8609 s	47
Proposed Q-learning in the presence of 2 dynamic Obstacles	348.6523 s	59
Proposed Q-learning in the presence of 4 dynamic Obstacles	367.6854 s	73

**Table 3:** Training parameters.

lterations	Batch Size	Discount Factory	Initial Learning Rate
120,000	32	0.95	0.000008

## Data Availability

Data are contained within the article.
